# The Role of Titanium Dioxide on the Hydration of Portland Cement: A Combined NMR and Ultrasonic Study

**DOI:** 10.3390/molecules25225364

**Published:** 2020-11-17

**Authors:** George Diamantopoulos, Marios Katsiotis, Michael Fardis, Ioannis Karatasios, Saeed Alhassan, Marina Karagianni, George Papavassiliou, Jamal Hassan

**Affiliations:** 1 Department of Physics, Khalifa University, Abu Dhabi 127788, UAE; g.diamantopoulos@inn.demokritos.gr; 2 Institute of Nanoscience and Nanotechnology, NCSR Demokritos, 15310 Aghia Paraskevi, Attikis, Greece; mikappa1@gmail.com (M.K.); m.fardis@inn.demokritos.gr (M.F.); i.karatasios@inn.demokritos.gr (I.K.); m.karagianni@inn.demokritos.gr (M.K.); g.papavassiliou@inn.demokritos.gr (G.P.); 3 Department of Chemical Engineering, Khalifa University, Abu Dhabi 127788, UAE; saeed.alkhazraji@ku.ac.ae

**Keywords:** cement hydration, titanium dioxide TiO_2_, NMR, ultrasonic, calorimetry

## Abstract

Titanium dioxide (TiO_2_) is an excellent photocatalytic material that imparts biocidal, self-cleaning and smog-abating functionalities when added to cement-based materials. The presence of TiO_2_ influences the hydration process of cement and the development of its internal structure. In this article, the hydration process and development of a pore network of cement pastes containing different ratios of TiO_2_ were studied using two noninvasive techniques (ultrasonic and NMR). Ultrasonic results show that the addition of TiO_2_ enhances the mechanical properties of cement paste during early-age hydration, while an opposite behavior is observed at later hydration stages. Calorimetry and NMR spin–lattice relaxation time T_1_ results indicated an enhancement of the early hydration reaction. Two pore size distributions were identified to evolve separately from each other during hydration: small gel pores exhibiting short T_1_ values and large capillary pores with long T_1_ values. During early hydration times, TiO_2_ is shown to accelerate the formation of cement gel and reduce capillary porosity. At late hydration times, TiO_2_ appears to hamper hydration, presumably by hindering the transfer of water molecules to access unhydrated cement grains. The percolation thresholds were calculated from both NMR and ultrasonic data with a good agreement between both results.

## 1. Introduction

Titanium dioxide (TiO_2_) has been studied for potential applications, notably as a white pigment and in hydrolysis [1] and electricity production [2], as well as an additive in construction materials (cement, concrete, tiles and windows) for its sterilizing, deodorizing and antifouling properties [3,4,5,6,7,8]. TiO_2_ integrated into construction materials effectively decomposes or deactivates volatile organic compounds, removing bacteria and other harmful agents. Cement-based, self-cleaning construction materials can play a major role in achieving clean air conditions in modern urban environments. Accordingly, it is of critical importance to characterize the structural properties and hydration kinetics of these materials to improve their mechanical performance.

Different, nondestructive methods are used to investigate the hydration of cement-based materials. The authors of [9,10] provide an overall view of these techniques. NMR has the advantage of nuclear-spin selectivity, where only one nuclear-spin isotope is detected at a time. The resulting resonances provide information on the local structure and dynamic effects. NMR allows studying the development of cement microstructures and kinetics in real-time during cement hydration, even at the earliest hydration times. Until now, several NMR techniques have been reported to provide valuable information on the porosity, pore size distribution and hydration kinetics of cement pastes. Such techniques include NMR cryoporometry [11,12], imaging [13,14,15] and diffusion studies [16,17,18,19]. The most widely used technique to study the porosity and hydration of cement pastes is proton (^1^H) NMR relaxometry [20,21,22,23]. In this method, the molecular motion and chemical and physical environments of water molecules are probed continuously by measuring the ^1^H nuclear-spin–lattice and spin–spin relaxation times of hydrogen nuclei. Ultrasonic wave velocity measurements similarly provide an excellent nondestructive tool to continuously monitor the evolution of the solid matrix as cement hydrates from the initial fluid state to the final solid configuration [24,25]. Measuring the speed of ultrasonic waves propagating through the cement slurry allows for in situ monitoring of hydration dynamics and determination of elastic properties [26,27,28].

When additives with hydrophilic properties, such as TiO_2_, are added to a cement paste, the hydration process and pore size development are affected in multiple ways [29,30]. It is known that the surface of fine fillers provides additional sites for the nucleation of C–S–H, accelerating the hydration reaction by reducing the energy barrier [31]. The effectiveness of this catalytic effect depends on the dosage and fineness of the nanoparticles. For TiO_2_ nanoparticles, their addition to cement can result in decreased water permeability and improved durability properties, such as chloride penetration and capillary adsorption [29]. It has been reported that the addition of TiO_2_ nanoparticles to ordinary Portland cement results in an accelerating effect of early cement hydration, directly proportional to particle/agglomerate size [32]. TiO_2_ nanoparticles acquire a negatively charged surface in the early-stage “ionic soup” of hydrating cement, balanced by increasing Ca^+2^ concentrations. Agglomeration is promoted via ion-ion correlations, also observed in C–S–H gel particles [33]. It is known that adding TiO_2_ to cementitious materials enhances the mechanical properties of the cement in the early age of hydration. Enhanced mechanical properties for cementitious materials doped with TiO_2_ are also reported at the late stage of hydration [32,34,35,36,37,38,39]. In a recent review by Rashad [40], the optimum percentages of TiO_2_ in cementitious materials at which mechanical properties enhanced were summarized as 1–4% for concrete, 2–10% in mortars and up to 10% in pastes. Other studies reported opposite results, where the mechanical properties of cementitious materials doped with TiO_2_ decreased at later stages of hydration [41,42,43]. The effect of adding TiO_2_ in cement hydration and hardening, as well as the effect on the mechanical properties at late hydration times, are not fully clarified. This is considerably important for the development of new construction materials that incorporate TiO_2_ for its sterilizing effects but less likely for its photocatalytic properties where a more practical approach would be the application of coatings to the exterior surface of the structures.

In this article, results from both NMR and ultrasonic velocity measurements on the effect of TiO_2_ on the hydration of Portland cement are presented. NMR ^1^H spin–lattice relaxation and diffusion data are recorded. These measurements monitor the dynamics of water molecules confined in cement pores and their interaction with the pore surface.

## 2. Experimental

### 2.1. Ultrasonic Section

In bulk solid materials, ultrasonic waves propagate mainly in two modes: shear and longitudinal waves. In cement, shear waves are expected to propagate only after C–S–H cement gel first percolates (i.e., at cement setting time) [24]. On the other hand, longitudinal waves propagate through the initial suspension. As the hydration procedure continues, the system becomes increasingly rigid and develops a solid matrix of pores filled with water. The longitudinal *V*_L_ and shear *V*_s_ wave velocities increase as both the bulk and shear moduli increase rapidly in value. *V*_L_ is related to the constrained modulus [44], *M =* 𝜌 *V*_L_^2^, where ρis the density of the sample. By monitoring the evolution of *V*_L_ during the hydration process and the evolution of the cement paste density, it is possible to deduce the evolution of the M modulus of the cement paste as a function of the hydration time. During the propagation of the signal through a material, its spectrum deforms and experiences high damping [25,26,45,46].

### 2.2. NMR Section: Spin-Lattice Relaxation Time

In this study, ^1^H-NMR spin–lattice relaxation time *T*_1_ measurements are used to obtain information on the hydration process in a nondestructive manner. *T*_1_ is determined by the water/solid interface of the cement system and the development of the pore network. The relaxation rate, 1*/T*_1_, of mobile water molecules increases near a liquid-solid interface due to the exchange between the free and bonded water and the presence of paramagnetic sites on the solid surface [13]. In the fast-exchange approximation:

(1) $$\frac{1}{T_{1}}=\frac{1}{T_{1b}}\left(p\right)+\frac{1}{T_{1f}}\left(1-p\right)$$

where the subscripts b and f refer to the “bonded” water near the pore surface and “free” water, respectively; p is the fraction of bonded water molecules at pore surfaces [22,47]. For spherical pores with mean radius *r*, assuming bonded water molecules form a layer of thickness *ε* [23],

(2) $$p=\frac{3\varepsilon }{r}=\frac{\varepsilon S}{V}$$

where *S/V* is the pore surface area to volume ratio. As $\frac{1}{T_{1f}}\ll \frac{1}{T_{1b}}$, due to the presence of paramagnetic sites on the solid surface, the overall relaxation rate depends linearly on the *S/V* of the pores.

(3) $$\frac{1}{T_{1}}=\frac{1}{T_{1b}}\left(\frac{pS}{V}\right)$$

In cement and other complex porous materials, *T*_1_ spreads over a wide distribution of relaxation times due to the existence of pore sizes ranging from nanometers to micrometers. During cement hydration, pore networks develop within the cement matrix. The nuclear magnetization in a saturation recovery experiment can be expressed as [23]:

(4) $$R\left(t\right)=\frac{M_{o}-M\left(t\right)}{M_{o}}\;\int_{0}^{\infty }g\;\left(T_{1}\right)\exp\left(\;-\frac{t}{T_{1}}\right)\;\;dT_{1}$$

where *R*(*t*) is the proton magnetization recovery function, *M*_0_ is the magnitude of the magnetization at equilibrium and *M*(*t*) is the observed magnetization as a function of time *t*. Here, *g*(*T*_1_) is the *T*_1_ distribution function, which can be resolved by means of an inverse Laplace transform [23], unveiling important information on the porous microstructure in the hardened material.

There are mainly three different “water groups” in hydrating cement pastes, which can be monitored by T_1_^1^H-NMR relaxometry. First, water chemically bound to OH groups (portlandite, gypsum and ettringite) exhibits a restricted motion, characterized by long *T*_1_ (> 100 ms) and very short spin-spin *T*_2_ (≈10 µs) relaxation times. In this study, this water group is intentionally excluded from the data acquisition by setting the experimental time window for the NMR measurements accordingly. Second, mobile water is incorporated into the C–S–H phase and located in the restricted volume of the gel pores. The relaxation is dominated by the pore–surface interactions, resulting in short *T*_1_ and *T*_2_ values (0.5–1.0 ms). Monitoring the gel pore water is of primary importance, as it controls the viscoelastic response of C–S–H gel to mechanical loading and relative humidity changes (drying shrinkage). Third, water is trapped inside capillary pores (3–50 nm) and microcracks of the hydrating cement pastes, with considerably higher relaxation times (~5–10 ms) [48,49], albeit lower compared to bulk water (~2 s).

### 2.3. NMR Section: Spin-Spin Relaxation Time and Diffusion Measurements

Measuring the water self-diffusion coefficient (*D*) is important in studying cement, as it is directly related to hydration and the development of the gel matrix. *D* is directly connected to water permeability and thus to the durability and aging properties of cement. The conventional NMR method for measuring *D* is to monitor the ^1^H-NMR spin echo decay in a constant linear magnetic field gradient. For the isotropic diffusion, the NMR data can be fitted to the relation [19]:

(5) $$M\left(2\tau \right)=M_{o}\;\exp\left(\frac{2\tau }{T_{2}}-\frac{2}{3}\;\gamma^{2}\;D\;G^{2}\;\tau^{3}\right)$$

where *γ* = 26.7522 × 10^7^ rad s^-1^T^−1^ is the gyromagnetic ratio for proton, and *G* is the magnetic field gradient. The linearly exponential part of the decay corresponds to *T_2_* and the cubic exponential decay corresponds to dephasing due to the presence of the magnetic field gradient. However, water molecules in porous systems do not diffuse freely due to pore confinements. Therefore, the dephasing part of the spin echo deviates from the previous equation [18,19,50]. To describe the dephasing behavior, two length scales need to be compared [50,51]: structural length *l_S_* (=*V/S*, for spherical pores) and dephasing length $l_{G}$, i.e., the distance a particle must travel to dephase by a full cycle in the magnetic field gradient. If the diffusion length $I_{D}(=\sqrt{6D\tau )}<l_{S}\;or<l_{G}$, water molecules diffuse freely in the porous matrix and Equation (5) is valid. However, if $l_{S}<l_{D}$, the magnetization decay is in the so-called motional averaging regime [50,51], and the dephasing part of the spin echo decay is characterized by a single exponential law. For the case of spherical pores [52,53]:

(6) $$M\left(2\tau \right)=M_{o}\exp\left[-\left(\frac{8}{175}\frac{\gamma^{2}\;G^{2}\;R^{4}}{D}\right)\;2\tau \;\right]$$

On the other hand, if $l_{G}<l_{S}\;and<I_{D}$, the magnetization decay is in the so-called localization regime [50], and the following expression applies:

(7) $$M\left(2\tau \right)=M_{o}\exp\left[-1.02\left({\left(\gamma \;G\right)}^{\frac{2}{3}}\;D^{\frac{1}{3}}\right)\;2\tau \;\right]$$

The above is applicable when the magnetic field gradient is very strong and water molecules have already dephased significantly before they reach the pore walls.

### 2.4. Materials

White cement (CEM II-42.5) was provided by Lafarge–Heracles (Greece) and TiO_2_ (P-25 Aeroxide) was purchased from Degussa. White cement was selected because of its low iron oxide content, which causes line shape broadening in NMR experiments due to magnetic susceptibility effects. According to the manufacturer, P-25 TiO_2_ contains 70% anatase and 30% rutile (*w/w*), with average grain sizes of 21 nm and a specific surface area of 50 m^2^/g. Four cement paste mixtures were prepared, namely C100, C97T3, C93T7 and C85T15. The number at the end of the doped sample names refers to the weight percentage of TiO_2_ that replaced equal amounts of cement (3, 7 and 15% *w/w*, respectively). The water-to-cement ratio (*w/c*) was kept constant for all samples at *w/c* = 0.40. The percentages were chosen based on expansion measurements by Flow Table. The sample with 15% titania (C85T15) has a *w/c* ratio of 0.40, which is also its normal plasticity water, thus ensuring its workability. All other percentages are roughly selected by dividing the percentage of titania. Regarding sample preparation, cement and TiO_2_ were initially mixed together at the appropriate weight ratio of each specimen and stirred at a low mixer speed for 10 min to ensure excellent dispersion of TiO_2_ in cement. Mixing with distilled water was performed according to the procedure described in the EN 196-1 standard [54]. For the ultrasonic experiments, each sample was cast in a Plexiglas cubic mold (10 × 10 × 10 cm) immediately after mixing with water. The thickness of the Plexiglas wall at the position of contact with the ultrasonic transducers was 0.25 cm. Care was taken to ensure constant pressure on the transducers attached on opposite sides of the mold by the use of springs. The quality of the contact was assured by the application of lubricant between the transducers and the Plexiglas mold. Samples were saturated with distilled water after 6 h of casting and the open top of the molds was membrane-sealed. With this procedure, shrinkage was minimized, and good contact between the Plexiglas wall and the curing cement was ensured to avoid sound attenuation and signal loss. Ultrasonic measurements were conducted with a commercial ultrasonic pulse generator (GE USM 23) driving two identical transducers of nominal frequency (500 kHz). The waveforms were recorded with an A/D converter using LabView home-built software. All experiments were performed at room temperature for a minimum time period of 80 days and conducted four times. The values of the ultrasonic velocity measurements provided here are the mean values of the four experiments. The estimated uncertainty for the ultrasonic velocity measurements was ± 30 m/s (calculated as ½ (V_max_ − V_min_), where V_max_ and V_min_ are the maximum and minimum velocity values as measured from the experiments). For the NMR experiments, samples were taken from the same mixtures used in the ultrasonic measurements to ensure identical preparation conditions. Immediately after mixing with water, the samples were sealed into NMR glass tubes (9 mm in diameter and 30 mm in length) using a Parafilm^®^ membrane to minimize the evaporation of water. *^1^H-T*_1_ experiments were conducted using a home-built circular Halbach array magnet, suitable for low-field NMR measurements [55]. The field at the magnet center was 0.29 T, corresponding to a proton resonance frequency. The magnet was coupled to a broadband spectrometer operating in the frequency range of 5–800 MHz. *T*_1_ was measured using a standard saturation recovery technique ((*π*/2) − *t* − (*π*/2) − *τ* − (*π*)) with the interpulse delay, *t*, ranging between 100 μs and 6 s on a logarithmic scale. The signal was detected by the common Hahn echo pulse sequence with a *τ* value of 60 μs. All experiments were performed at room temperature and the hydration process for each sample was monitored for 28 days. Time intervals between successive experiments ranged from minutes and several hours in the initial hydration stage up to full days at the later hydration stages. At the early stage of hydration, *T*_1_ was characterized by a single exponential function. However, with progressive hydration, a multiexponential behavior 12.1718 developed, which was resolved by means of an inverse Laplace transform [55]. The numerical Laplace inversion of the ^1^H- of 12.1718 MHz NMR saturation recovery curves was obtained using a modified CONTIN algorithm [56], which was constrained to a positive output for 30 logarithmically distributed points between *T*_1min_ = 0.01 ms and *T*_1max_ = 10.000 ms.

Water diffusion experiments were also conducted in the same Halbach magnet with a magnetic field gradient of 1.03 T m^–1^. Measurements were carried out using the Hahn echo pulse sequence ((*π*/2) − *τ* − (*π*) − *τ* − echo) with the interpulse delay, τ, ranging from 20 to 8 ms. All experiments were performed at room temperature and the hydration process for each sample was monitored for 300 h with time intervals between successive experiments ranging from minutes to several hours. The uncertainty in the T_1_ values was estimated at ±0.01 ms.

From the same batch mixtures that were used for ultrasonic and NMR analyses, an additional set of cement-TiO_2_ specimens were prepared to measure microstructural properties during hydration. Each specimen was set in prismatic molds (20 × 20 × 80 mm) and left to hydrate at a relative humidity of 98 ± 2% and a temperature of 21 ± 1 °C inside a curing chamber. The molds were membrane-sealed and covered with glass sheets to avoid water evaporation. After two days, the specimens were removed from the molds. These prismatic specimens were used to measure density at progressing hydration ages using the standard Archimedes method with water. All specimens were examined during the first 28 days of hydration with scanning electron microscopy (SEM) using a FEI Quanta Inspect coupled with an energy-dispersive spectroscopy (EDS) unit. Specimens were vacuum dried using ethanol and diethyl ether prior to analysis and gold-coated. Standard Vicat measurements were performed and isothermal calorimetry measurements were carried out in an I-CAL 2000 HPC, Calmetrix at 20.0 ± 0.5 °C. The hydration of the mixtures was monitored by simultaneous differential thermal analysis and thermogravimetry (DTA/TG) in a Perkin Elmer Pyris 2000 thermal analyzer [57].

## 3. Results and Discussion

### 3.1. Vicat, Isothermal Calorimetry and DTA/TG Measurements

**Figure 1:**
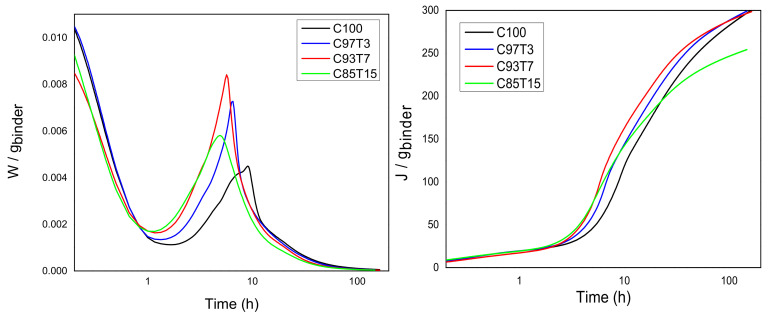
Isothermal calorimetry measurement. Power versus hydration time and heat release versus hydration time divided by the total solid mass.

Figure 1 exhibits the normalized rate of hydration (per gram of cement + TiO_2_) for all mixtures, according to isothermal calorimetry measurements performed for the first seven days. Note that the *w/c* ratio is the same for all mixtures.

These calorimetry data show that the peak heights were increased with the addition of the inert TiO_2_ nanoparticles, thus the addition of TiO_2_ greatly affects the early-age hydration. The TiO_2_-doped samples’ heat release curves around 5 h are shifted to the left, indicating the early-age acceleration effect of TiO_2_. Although Titania is inert, the rate of reaction of the clinker component is enhanced. The main peak is higher for the samples with 7% titania, while for all doped samples, it is higher than the reference sample, and the acceleration slope is steeper for all the doped samples compared to the reference sample. The cumulative heat of the C85T15 sample falls lower than the reference sample after approximately 24 h. Considering that *J* in Figure 1 is referred to as the extent of the hydration process, the differentiation of mixture C85T15 is attributed to the sorter retardation/deceleration period and the beginning of the long-term reactions at earlier stages. Overall, the full curve of C85T15 is shifted to the left.

Standard Vicat measurements were performed in all mixtures for determining the initial and final setting time (see Table 1 in Section 3.5), using the same amount of water.

The setting time determined by different methods and the hydration process are presented and discussed in Table 2 (Section 3.5).

Based on the peaks appeared in the DTA curves, the total amount of chemically bound water (attributed to the hydrated C–S–H and C–A–H phases) was calculated gravimetrically (TG%) at different setting periods in the range of 75–320 °C [58,59]. Samples were heated in an air atmosphere in three steps: from 25 to 60 °C, using a heating rate of 5 °C/min; a hold-step at 60 °C for 1 h (aiming to remove any physically adsorbed water; and heating up to 400 °C with a rate of 10 °C/min. The amount of chemically bound water was compared to that calculated in a reference cement specimen (C100R) after setting for two years at the same curing conditions.

**Figure 2:**
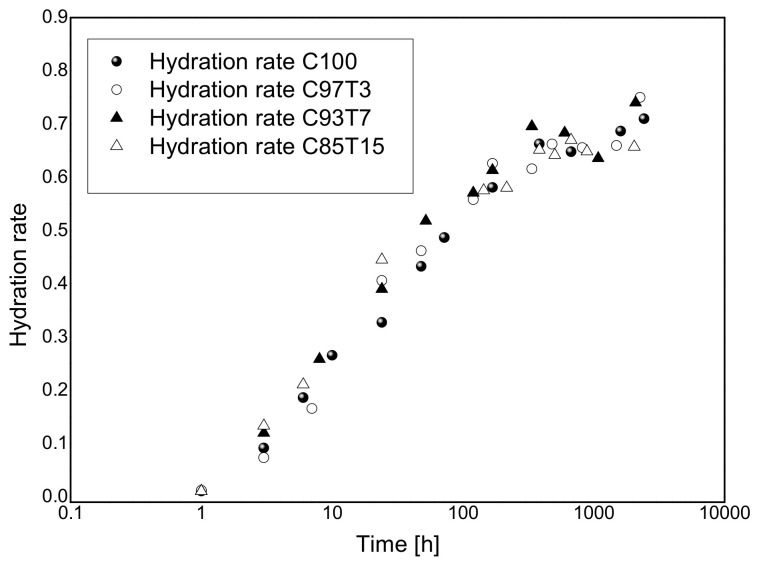
Normalized hydration rates of the four samples with respect to the fully hydrated C100R sample.

The hydration rate (R_H_) of each different cement-TiO_2_ mixture was calculated (Figure 2) by dividing the amount of bound water at different setting periods by the amount of bound water in the fully hydrated cement (C100R) [60].

### 3.2. Ultrasonic Experiments

**Figure 3:**
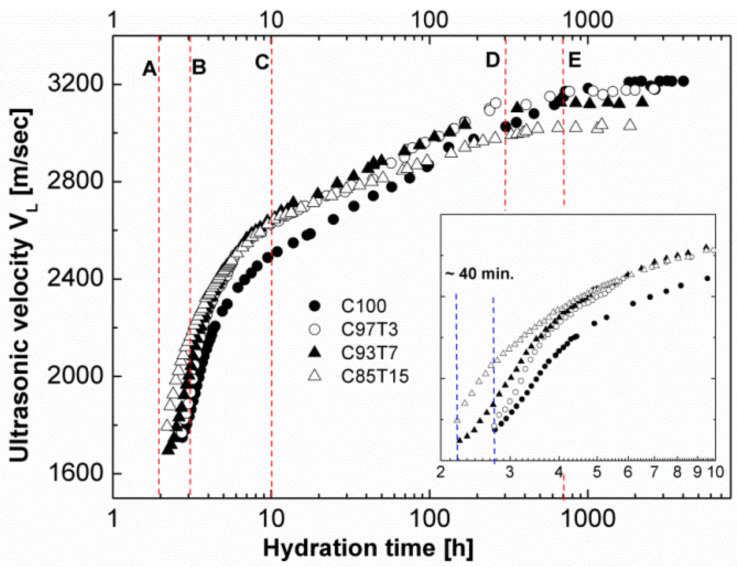
Ultrasonic longitudinal velocity versus hydration time for the samples used in this study. The inset presents the data during the first ten hours of hydration time. The vertical dashed lines are presented for clarification and to indicate the different hydration periods as discussed within the text.

The hydration process for all samples was monitored by means of continuously measuring the longitudinal ultrasonic wave velocity as a function of hydration time. Figure 3 demonstrates the longitudinal ultrasonic velocity *V*_L_ as a function of hydration time. As seen from Figure 3, the hydration periods of cement [27,61,62] are distinguished in the time evolution of *V*_L_ except for the “dormant period”, which occurs at the early time (below 2 h) of hydration, where cement paste behaves as a liquid suspension of particles. In our measurements, no longitudinal velocity signals were observed at the dormant period (left region of the dashed line A, Figure 3) as entrapped air led to strong attenuation of the longitudinal waves [25,26,62]. Air bubbles within the binder blocked the higher frequencies at the early-age, allowing them to transmit at a later-age [26,45,63,64,65]. The evolution of the frequency follows the evolution of the ultrasonic velocity and asymptotically reaches the carrier frequency at later times. Between ~2 and 3 h of hydration (dashed lines A and B), ultrasonic waves become detectable and *V*_L_ increases rapidly with hydration time.

Samples with higher TiO_2_ content exhibited higher *V*_L_ values. The *V*_L_ value of the sample with the highest TiO_2_ content, C85T15, was recorded approximately 40 min earlier compared to the reference sample, C100 (notice the signal between the two blue lines in the inset of the figure). This indicates that substituting cement with TiO_2_ causes a strong acceleration of the early hydration. In the “acceleration period” (~3–0 h, lines B and C), an increased hydration rate is observed for all the samples with the lowest *V*_L_ values for the reference sample. The rapid growth of cement gel in this period facilitates solid pathways, which enables the transmission and detection of longitudinal waves [28,45]. After approximately 10 h of hydration, the “deceleration period” began with a slower increase in *V*_L_ for all samples compared to the acceleration period. This period lasted for up to ~300 h of hydration for the TiO_2_-containing samples (between lines C and D) and up to ~ 700 h for the reference sample (between lines C and E). The data show a nonlinear behavior for the TiO_2_-containing specimens and a linear behavior for the reference sample, a characteristic of adding fine aggregates to cement [66,67,68,69,70]. The addition of fine aggregates accelerates the evolution of the microstructure [51]. Therefore, additives such as TiO_2_ caused a reduction of the distances among solid particles, and smaller amounts of hydration products are needed to form the first connection path for the ultrasonic signal to transmit. This “filler effect” is also observed in the SEM images of the samples and presented later in this section (Figure 8). A further observation of the data at the end of this period indicates that the *V*_L_ value of the reference sample overpassed the *V*_L_ values of the other samples, indicating its solid paths became fully developed. This is opposite to what was observed at the early stage of hydration (between the two blue lines).

Furthermore, all samples reach a saturation value in their ultrasound velocity after 1000 h. The saturation values from Figure 3 are calculated as 3018 m/s for sample C85T15, 3123 m/s for sample C93T7, 3169 m/s for sample C97T3 and 3207 m/s for sample C100.

**Figure 4:**
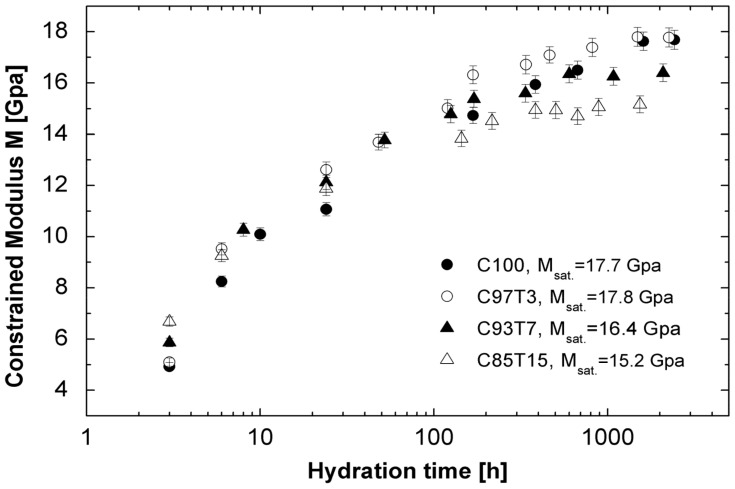
Evolution of constrained modulus *M* for the samples versus hydration time.

These values are inversely proportional to the amount of TiO_2_ in the sample, indicating the reduction of the constrained modulus with increasing TiO_2_ content in cement. In this connection, the constrained modulus (*M =* 𝜌*V*_L_^2^) of the samples is calculated, and the results are presented in Figure 4.

The saturation values of *M*_sat_ are 15.2, 16.4, 17.7 and 17.8 Gpa for C85T15, C93T7, C100 and C97T3, respectively. It is known that TiO_2_ is added to construction materials (cement, concrete, tiles and windows) for its sterilizing, deodorizing and antifouling properties [3,4,5,6,7,8]. The results of Figure 4 show that if too much TiO_2_ (15%, as in C85T15) is used, the mechanical properties of the material will be affected.

### 3.3. ^1^H-Spin–Lattice Relaxation Time (T_1_) Measurements

**Figure 5:**
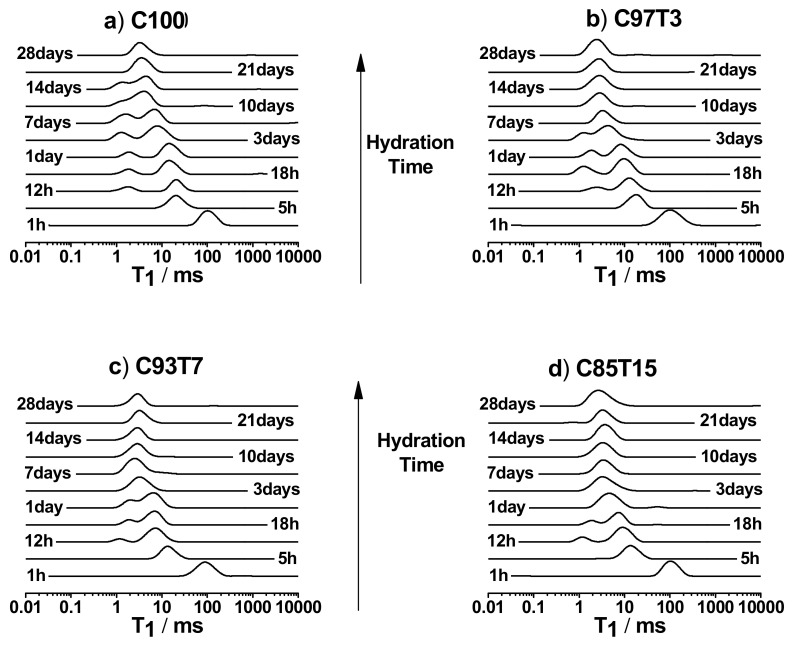
NMR spin–lattice relaxation T_1_ distribution profile versus hydration time obtained from the inverse Laplace transform of the NMR data for (**a**) C100, (**b**) C97T3, (**c**) C93T7 and (**d**) C85T15, samples. Note the change of the T_1_ profile from one, two, to one component at short, intermediate and long hydration times, respectively.

The hydration process for the samples was monitored by measuring *^1^H-T_1_*, which is less liable to artifacts caused by water molecule diffusion in magnetic field in-homogeneities. Figure 5 shows *T*_1_ distribution profiles, obtained by the inverse Laplace transform [55], as a function of hydration time. For all samples and at early hours of hydration, the magnetization of water protons relaxes uniformly due to the fast exchange between water spins in the various environments, represented by a single *T*_1_ component [20,71,72]. *T*_1_ for this peak is ~ 100 ms. With proceeding hydration, a growing number of hydration products develop and the paste becomes rigid. The surface area of the pore network increases, which causes a reduction of *T*_1_, as predicted by Equation (3). Monitoring the change in *T*_1_ allows the observation of the evolution of hydration and the growth of the pore structure of cement. At approximately 12 h of hydration, *T*_1_ distribution splits into two components. The short and long *T*_1_ peaks are attributed to water in gel pores and large capillary pores, respectively [55,73]. The observation of these two components indicates the formation of both gel and capillary pores, with different pore geometries reflected by their *T*_1_ values. At longer hydration times, the cement paste hardens and a further reduction of *T*_1_ is observed, as a result of increasing pore surface areas according to Equation (3). Beyond 12 h of hydration, the peak assigned to the gel pore (short *T*_1_) grew larger in area while the capillary peak (long *T*_1_) decreased slightly, providing a measure of the change of the two pore populations. The two *T*_1_ peaks are visible over different time intervals depending on the percentage of the TiO_2_ in each sample (with the reference sample lasting longer). The two *T*_1_ peaks are visible from 12 h for all samples and retain until 18 h, 1 day, 3 days and 14 days for C85T15, C93T7, C97T3 and C100, respectively. Beyond these times, both peaks merge into one. This behavior can be related to the deceleration period of the samples, as deduced from ultrasonic results (Figure 3), where the deceleration period lasted longer for the reference sample. Another observation on the two peaks of *T*_1_ in Figure 5 is that the gel pore peak (short *T*_1_) attains relatively higher initial values in the TiO_2_-containing samples compared to the reference sample, while it subsequently shifts to lower *T*_1_ values with progressing hydration. This might be due to the creation of an initial “more open” gel pore structure.

**Figure 6:**
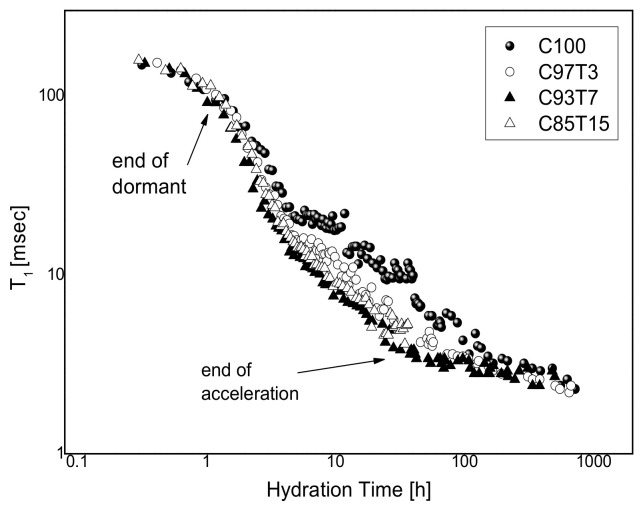
^1^*H-T*_1_ NMR measurements for all four samples versus hydration time.

Figure 6 shows the evolution of the average *T_1_* as a function of hydration time for the four cement samples. A formation of a “shoulder” in the T1 versus hydration time curve is observed at about 5 h for both C97T3 and C100 samples but is more visible in the C100 sample. The formation of this shoulder is known to relate with the *w/c* ratio and is more pronounced at a higher *w/c* ratio [12], related to a second water release originating from the melting of solid substructures, mainly ettringite crystals into monosulfate [41,42]. Although the samples used in this study have the same *w/c* ratios, C93T7 and C85T15 do not exhibit such a shoulder contrary to C100 and C97T3 samples. This behavior can only be attributed to the role of titania in withholding water, making the samples with a larger titian content not exhibit such a shoulder curve.

**Figure 7:**
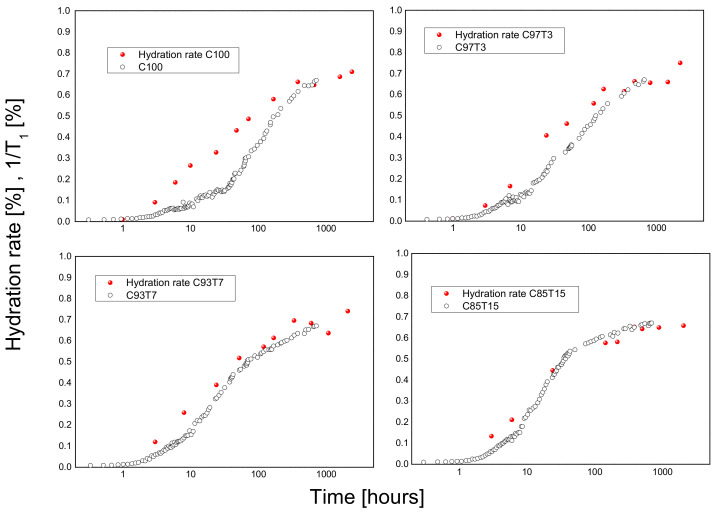
Hydration rates and normalized mean relaxation rates ${\left[\frac{1}{T_{1}}\right]}_{norm}$ versus hydration time for all four samples calculated by DTA/TG measurements.

The ratios of the mean relaxation rates 1/T1norm=1T11T1max versus hydration time are obtained using the procedure described in [55]. This quantity is the average relaxation rate and is a measure of the development of the fine porous system by the increase of the total surface area of the porous system. The increase of the total surface area is related to the formation of hydration products and follows the hydration rate of the samples [17,39,40], regarded as a well-defined parameter for a heterogeneous multiphase system such as cement (gel and capillary pores) [59]. Figure 7 shows the connection between the hydration rates derived from DTA/TG measurements and the normalized NMR 1/T_1_ rates. For the samples C100 and C97T3, there is a significant deviation between DGA/TA and NMR results. This deviation is related to the formation of the shoulder in the T_1_ measurements in those samples (Figure 6). This behavior could be explained by the hydrophilicity of TiO_2_ nanoadditives and the consequent stronger van der Waals forces between water and titania. This has a consequent effect on the water molecules’ mobility (considerably reduced by increasing the amount of TiO_2_) and the resulting ^1^H T_1_ NMR measurements. The TG measurements are affected, since the fast heating rate (10 °C/min) cannot overcome the strong hydrophilic forces between water molecules, resulting in a decrease of the hydration related water and a better fit to NMR results. On the other hand, T_1_ NMR monitors all mobile water molecules both in gel and capillary pores. For the samples C93T7 and C85T15, which do not manifest a shoulder in the T_1_ results, a very good coherence between these two techniques is shown.

An increase in relaxation rates is observed for the higher-doped samples. Increasing relaxation rates correspond to an increase in the pore surface areas, as expected by Equation (3). The enhancement is believed to be due to two reasons: (1) TiO_2_ grains promotes C–S–H gel production and the grains act as nucleation centers for cement gel growth, causing an increase in the short T_1_ peak area versus hydration time (Figure 5); (2) TiO_2_ speeds up the rupture of the gelatinous coating created around cement grains [31,74], therefore the dormant period is shorter for the doped samples, as seen at the beginning of the hydration time of Figure 6.

**Figure 8:**
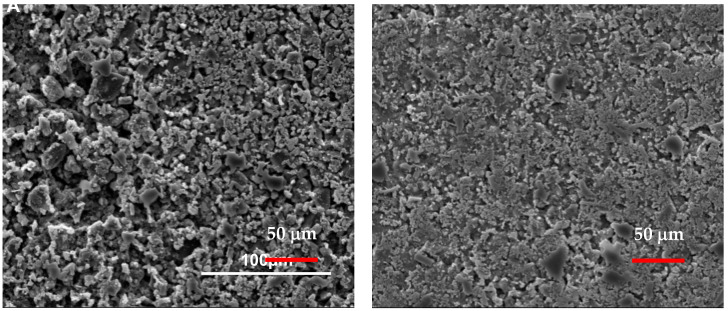
Scanning electron microscopy (SEM) images for specimens C100 (left) and C93T7 (right) after two days of hydration. The 7% TiO_2_ (right image) sample exhibits a significantly denser internal structure compared to the reference sample (left image).

For the SEM images, the specimens were cast on freshly cleaved flat mica surfaces and left to cure for two days at 21 °C and 95% RH. Samples were then placed on SEM aluminum stubs and the mica surface was removed. Images were collected from the surface of each specimen exposed to mica. Figure 8 shows the SEM images of the reference sample, C100, and the 7% TiO_2_ sample, C93T7. The latter appears to have a denser and more packed structure compared to C100 due to the high production of early hydration products. This can also be related to the NMR results (shown in Figure 5; Figure 6), which revealed (from 1/*T*_1_) an increased production of cement gel in doped samples compared to those in the reference sample.

### 3.4. ^1^H-NMR Diffusion Measurements

**Figure 9:**
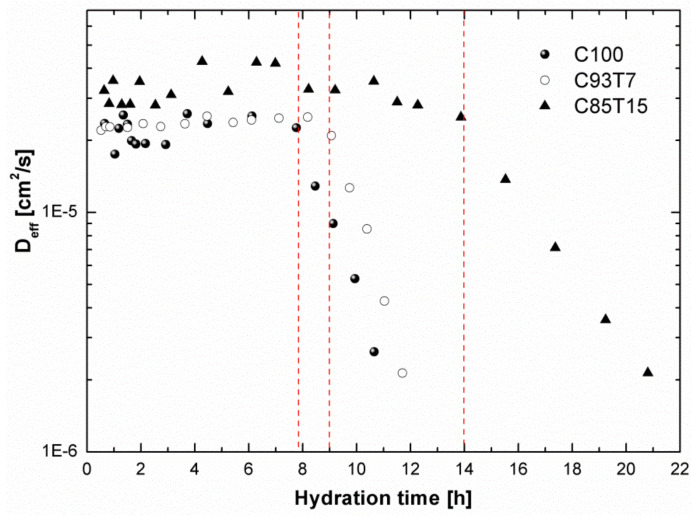
Self-diffusion coefficient, *D_eff_* versus hydration time obtained from NMR data by using Equations (5)–(7). The time at which *D_eff_* has decreased depends on the TiO_2_ concentration. For the 15% TiO_2_ sample, this started at a longer time compared to the other samples.

Water mobility inside the porous system of hardening cement was monitored through the evaluation of self-diffusion coefficient, *D_eff_*, as determined from the NMR diffusion data using Equations (5)–(7). The results are shown in Figure 9. At the beginning of the dormant period, *D_eff_* remains practically unchanged, with the TiO_2_-containing specimens exhibiting higher values. Going more into this period, a sharp decrease is seen in the coefficient value for all specimens. This indicates a restriction of water mobility as the pore system develops due to the increased production of cement gel. The decrease in *D_eff_* directly corresponds to an increase in the pore size area. It is interesting to note that the aforementioned decrease in *D_eff_* takes place after an amount of time proportional to TiO_2_ content, as depicted by the dotted lines in Figure 9 (at approximately 8, 9 and 14 h for C100, C93T7 and C85T15, respectively).

### 3.5. Correlation between NMR and Ultrasonic Results

**Figure 10:**
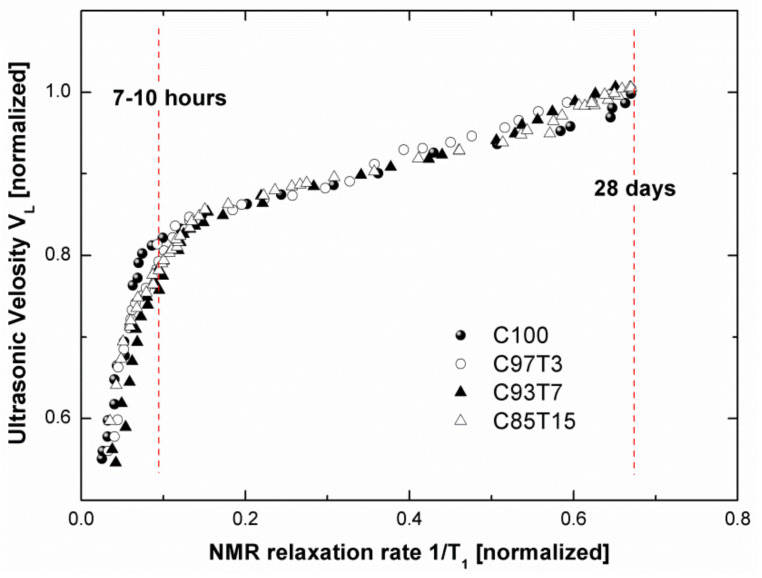
Correlation between ultrasonic longitudinal velocity and NMR relaxation rates. Both are normalized and the hydration times are taken as implicit parameters. Two distinct regions of linear correlations between V_L_ and 1/T_1_ are observed from the starting time until ~7–10 h and ~7–10 h until 28 days.

Ultrasonic longitudinal velocity *V*_L_ evolves with hydration time and is closely related to the formation of the pore network. On the other hand, NMR relaxation rate 1/T_1_ is proportional to the pore sizes through Equation (3) and provides a measure of the development of the fine pore system. Therefore, a correlation between these two properties can be made. Figure 9 presents the normalized *V*_L_ values versus the normalized 1/T_1_ values, considering hydration time as an implicit parameter. The 1/*T*_1_ values were normalized with respect to the 1/*T*_1_ value measured for the reference sample after a setting period of two years. *V*_L_ values were normalized with respect to the saturation values obtained from Figure 3. In Figure 9, two distinct regions of linear correlations between *V_L_* and *1/T_1_* can be observed. The first linear correlation appears immediately after the initial setting and continues for 7 to 10 h of hydration time. The second linear region appears directly after this period and continues for the entire time of the analysis (28 days). The steeper slope observed in the first region indicates that the ultrasonic technique is more sensitive than NMR during early hydration. *V*_L_ increases rapidly when the first solid pathways are formed, but water is still unhindered and pore size distributions (measured through 1/*T*_1_) are not yet distinguishable on the NMR time scale. With progressing hydration, water either reacts with cement or becomes confined within the porous system, resulting in an observable change in the NMR signal. In Figure 10, the slope of the second linear region is shifted toward the NMR 1/*T*_1_ axis, indicating that the NMR technique is more sensitive at later hydration times in detecting the development of the porous network. Accordingly, both techniques monitor the formation of hydration products through different yet complementary mechanisms.

**Figure 11:**
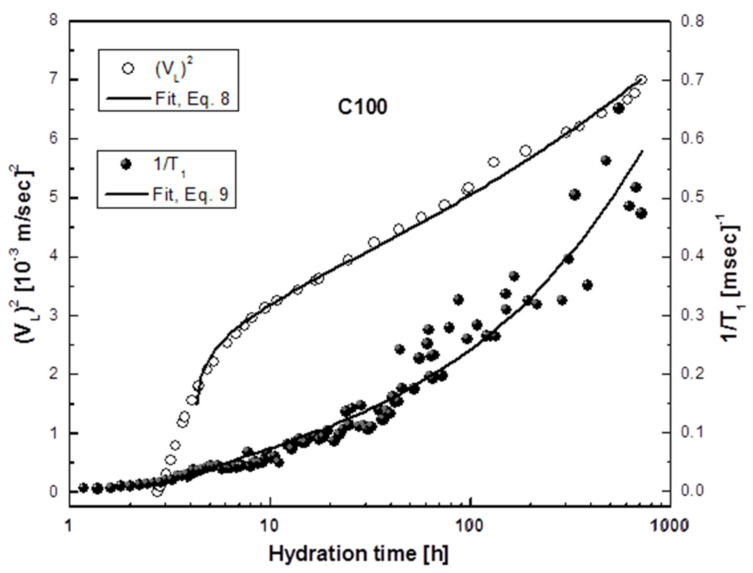
Fit of Equations (8) and (9) to the NMR data (black circles) and ultrasonic data (white circles) for the reference specimen C100 to obtain the percolation parameters 𝑝_c_ as described in the text.

As discussed previously, ^1^H-NMR experiments define the evolution of cement microstructure and the formation of a pore network over time by probing the water molecules and their interaction with the solid surface of the pores. ^1^H 1/*T*_1_ rates are a measure of hydration by probing the development and evolution of the pore network of the cement paste during the hydration process, as described by Equation (3). Ultrasonic measurements probe the development of the solid matrix by monitoring changes in the elastic modulus and density of the system through the transmitted ultrasonic waves. Percolation is a critical element in defining the performance of cement-based materials. To quantitatively characterize phase percolation, a complete understanding of microstructural changes in three dimensions is necessary. Experimentally, this can be challenging because commonly used techniques such as scanning electron microscopy or mercury intrusion porosimetry provide partial information or demand a significant amount of time to perform or to analyze [75]. On the other hand, the two techniques used in this study can be used to define percolation during cement hydration. In this context, each method defines the percolation of cement hydration in a different manner. As per ultrasonic methods, the percolation threshold can be defined as the point in time upon when the connectivity of the solid hydration products in the cement paste reaches a critical content, allowing for enhanced propagation of ultrasonic waves from that point onwards (acoustic percolation) [61,76]. From the NMR T_1_ relaxation perspective, the percolation threshold can be defined as the point in time when the interconnectivity of the pore network reaches a low critical value, following which T_1_ relaxation is controlled mostly by gel porosity (NMR relaxation percolation) [21,55,77]. By extending the power law equation used previously by Scherer et al. [76] (in ultrasonic, similar to Equation (8)) and NMR (Equation (9)), the percolation thresholds for *V*_L_ and 1/T_1_ can be calculated as:

(8) $$\left(V^{2}-V_{0}^{2}\right)\propto {\left(p-p_{c}\right)}^{\gamma }$$

(9) $$\left(\frac{1}{T_{1}}\right)-\left(\frac{1}{T_{10}}\right)\propto {\left(p-p_{c}\right)}^{\gamma }$$

where V_0_ and T_10_ are the initial values of V_L_ and T_1_, respectively. In order to determine the critical exponent 𝛾 and the percolation threshold, 𝑝_c_, ultrasonic and NMR data were fitted to the above equations, and a sample of the results is shown in Figure 11. From the fitted curves, the percolation parameters were obtained for all samples and presented in Table 1. The initial and final setting times are also presented in Table 1. These are obtained by Vicat measurements.

The threshold values obtained with ultrasonic, NMR and Vicat techniques (Table 1) are inversely proportional to the concentration of TiO_2_ in the samples. The ultrasonic parameters, p_c_, indicate a formation of solid pathways after ~5 h of hydration for the reference sample C100 and after ~3.5 h for the samples containing TiO_2_. Similarly, from the NMR part, the percolation threshold, p_c_, for pore critical low interconnectivity is reached after ~4 h for the reference sample and after ~3 h for the TiO_2_ samples. The initial setting times from the Vicat measurements correlate with the NMR percolation times (Columns 2 and 3 of Table 1), while the final setting times correlate with the ultrasonic percolation times (Columns 1 and 4 of Table 1).

Furthermore, in the following table, all the important hydration periods are presented, as detected from all the methods used. The start of the acceleration derived from the NMR measurements coincides excellently with the start of acceleration derived from the isothermal calorimetry measurements (Rows 2 and 8 of Table 2, bold). The end of the acceleration, as detected from the ultrasonic measurements, is verified from the main peak position of the isothermal calorimetry measurements (Rows 6 and 9 of Table 2, shaded).

## 4. Conclusions

The effect of titanium dioxide TiO_2_ in cement hydration has been demonstrated, using two noninvasive techniques (NMR and ultrasonic). Spin–lattice relaxation times *T*_1_ and diffusion measurements were used to monitor the dynamics of water molecules confined in cement pores and their interaction with the pore surface. Critical information on the role of TiO_2_ on the pore developments during hydration of cement paste was obtained. Ultrasonic wave velocity measurements provided information on bulk mechanical properties of cement in the presence of TiO_2_ and the evolution of the cement-solid matrix.

An acceleration of early hydration kinetics in TiO_2_-containing samples was observed with NMR, specifically as an enhancement in early hydration and C–S–H gel production. The existence of two pore reservoirs was also revealed: a small gel pore reservoir (short *T*_1_ component) and a large capillary pore reservoir (long *T*_1_ component), evolving separately from each other with the progress of hydration. The presence of TiO_2_ appeared to favor the formation of gel pores more than capillary pores, providing further proof of its role as a nanofiller.

Hydration rates derived from DTA/TG measurements and the normalized NMR 1/T_1_ rate were well correlated for the highly doped samples. Ultrasonic results showed that TiO_2_ enhances the mechanical properties of cement paste during early hydration. The ultrasonic signal was detected in earlier hydration time for samples with TiO_2_, an indication that the additive improved cement paste consistency. On the other hand, opposite behavior was observed at later hydration times, as the elastic properties (defined by the saturation values of the ultrasonic velocities) and the constrained modulus *M* of cement pastes (after 28 days of hydration) were inversely proportional to TiO_2_ content. At later stages, TiO_2_ appears to hamper hydration, presumably by hindering the transfer of water molecules to access unhydrated cement grains. Isothermal calorimetry measurements further support our conclusions.

The percolation threshold parameters were calculated using data of both techniques. These parameters are inversely proportional to the concentration of TiO_2_ in the samples. Using the ultrasonic methodology, the acoustic percolation threshold can be identified as the moment when solid pathways are first formed and sound propagation is enhanced. NMR detected the percolation threshold as the point when the interconnectivity of the pore network reaches a low critical value, after which T_1_ relaxation is controlled mostly by gel porosity. These values were well correlated with initial and final setting times determined by the Vicat method.

## Figures and Tables

**Table 1:** Percolation parameters (𝑝_c_) obtained from fitting NMR and ultrasonic data to Equations (8) and (9) and weighted standard deviation. Vicat measurements with uncertainty values.

Sample	Percolation Threshold, p_c_		Vicat Setting Times (h)	
	Ultrasonic/h	NMR/h	Initial	Final
C100	4.7 ± 0.08	3.6 ± 0.1	4.18 ± 0.02	4.88 ± 0.02
C97T3	4.3 ± 0.1	3.3 ± 0.1	3.66 ± 0.02	4.26 ± 0.02
C93T7	3.7 ± 0.2	2.8 ± 0.2	3.35 ± 0.02	3.80 ± 0.02
C85T15	3.2 ± 0.4	2.2 ± 0.3	2.78 ± 0.02	3.15 ± 0.02

**Table 2:** Periods of the hydration process for all four samples.

	Samples	C100	C97T3	C93T7	C85T15
NMR	**Start of acceleration (h)**	**1.63**	**1.57**	**1.20**	**1.08**
	Formation of “shoulder” (h)	4.88	4.71	-	-
	Start of diffusion (h)	89.0	43.0	29.0	23.0
UltraSounic	Initial detection of signal (h)	2.75	2.73	2.22	2.20
	End of acceleration (h)	8.8	6.0	4.9	3.9
	End of deceleration (h)	692	570	490	306
Calorimetry	**Start of acceleration (h)**	**1.67**	**1.37**	**1.26**	**1.06**
	End of acceleration/start of deceleration (h)	9.04	6.48	5.66	4.87

## References

[1] Park J.H., Kim S., Bard A.J. (2006). Novel carbon-doped TiO_2_ nanotube arrays with high aspect ratios for efficient solar water splitting. Nano Lett..

[2] Nazeeruddin M.K., Kay A., Rodicio I., Humphry-Baker R., Mueller E., Liska P., Vlachopoulos N., Graetzel M. (1993). Conversion of light to electricity by cis-X2bis(2,2′-bipyridyl-4,4′-dicarboxylate)ruthenium(II) charge-transfer sensitizers (X = Cl-, Br-, I-, CN-, and SCN-) on nanocrystalline titanium dioxide electrodes. J. Am. Chem. Soc..

[3] Parkin I.P., Palgrave R.G. (2005). Self-cleaning coatings. J. Mater. Chem..

[4] Lackhoff M., Prieto X., Nestle N., Dehn F., Niessner R. (2003). Photocatalytic activity of semiconductor-modified cement—Influence of semiconductor type and cement ageing. Appl. Catal. B-Environ..

[5] Hüsken G., Hunger M., Ballari M.M., Brouwers H.J.H., Bittnar Z., Bartos P.J.M., Nemecek J., Smilauer V., Zeman J. (2009). The effect of various process conditions on the photocatalytic degradation of NO. NICOM3.

[6] Fujishima A., Hashimoto K., Watanabe T. (1999). TiO_2_ Photocatalysis, Fundamentals and Applications.

[7] VVallée F., Ruot B., Bonafous L., Guillot L., Pimpinelli N., Cassar L., Strini A., Mapelli E., Schiavi L., Gobin C. Innovative self-cleaning and de-polluting facade surfaces. Proceedings of the CIB World Building Congress.

[8] Ballari M.M., Hunger M., Hüsken G., Brouwers H.J.H., Bittnar Z., Bartos P.J.M., Nemecek J., Smilauer V., Zeman J. (2009). Heterogeneous Photocatalysis Applied to Concrete Pavement for Air Remediation. NICOM3.

[9] (2002). Guidebook on Non-Destructive Testing of Concrete Structures.

[10] Jennings H.M., Bullard J.W., Thomas J.J., Andrade J.E., Chen J.J., Scherer G.W. (2008). Characterization and Modeling of Pores and Surfaces in Cement Paste: Correlations to Processing and Properties. J. Adv. Concr. Technol..

[11] Jehng J.-Y., Sprague D., Halperin W. (1996). Pore structure of hydrating cement paste by magnetic resonance relaxation analysis and freezing. Magn. Reson. Imaging.

[12] Boguszyńska J., Tritt-Goc J. (2004). 1H NMR Cryoporometry Study of the Melting Behavior of Water in White Cement. Z. Nat. A.

[13] Papavassiliou G., Milia F., Fardis M., Rumm R., Laganas E. (1993). ^1^H Nuclear Magnetic Resonance Imaging of Water Diffusion in Hardened Cement Pastes. J. Am. Ceram. Soc..

[14] Prado P.J., Balcom B.J., Beyea S.D., Bremner T.W., Armstrong R.L., Pishe R., Gratten-Bellew P.E. (1998). Spatially resolved relaxometry and pore size distribution by single-point MRI methods: Porous media calorimetry. J. Phys. D Appl. Phys..

[15] Jaffer S., Lemaire C., Hansson C., Peemoeller H. (2007). MRI: A complementary tool for imaging cement pastes. Cem. Concr. Res..

[16] Friedemann K., Stallmach F., Kärger J. (2006). NMR diffusion and relaxation studies during cement hydration—A non-destructive approach for clarification of the mechanism of internal post curing of cementitious materials. Cem. Concr. Res..

[17] Nestle N., Galvosas P., Kärger J. (2007). Liquid-phase self-diffusion in hydrating cement pastes—Results from NMR studies and perspectives for further research. Cem. Concr. Res..

[18] Neuman C. (1974). Spin echo of spins diffusing in a bounded medium. J. Chem. Phys..

[19] De Swiet T.M., Sen P.N. (1994). Decay of nuclear magnetization by bounded diffusion in a constant field gradient. J. Chem. Phys..

[20] Nestle N. (2004). A Simple Semiempiric Model for NMR Relaxometry Data of Hydrating Cement Pastes. Cem. Concr. Res..

[21] Papavassiliou G., Fardis M., Laganas E., Leventis A., Hassanien A., Milia F., Papageorgiou A., Chaniotakis E. (1997). Role of the surface morphology in cement gel growth dynamics: A combined nuclear magnetic resonance and atomic force microscopy study. J. Appl. Phys..

[22] Blinc R., Dolinsek J., Lahajnar G., Sepe A., Zupančič I., Zumer S., Milia F., Pintar M.M. (1988). Spin-Lattice Relaxation of Water in Cement Gels. Z. Nat. A.

[23] Laganas E., Papavassiliou G., Fardis M., Leventis A., Milia F., Chaniotakis E., Meletiou C. (1995). ^1^H Nuclear Magnetic Resonance Relaxation Measurements in Developing Porous Structures: A Study in Hydrating Cement. J. Appl. Phys..

[24] D’Angelo R., Plona T.J., Schwartz L.M., Coveney P. (1995). Ultrasonic measurements on hydrating cement slurries. Adv. Cem. Based Mater..

[25] Boumiz A., Vernet C., Tenoudji F.C. (1996). Mechanical properties of cement pastes and mortars at early ages: Evolution with time and degree of hydration. Adv. Cem. Based Mater..

[26] Sayers C., Dahlin A. (1993). Propagation of ultrasound through hydrating cement pastes at early times. Adv. Cem. Based Mater..

[27] Keating J., Hannant D.J., Hibbert A.P. (1989). Comparison of shear modulus and UPV techniques to measure the build-up of structure in fresh cement pastes used in oil well cementing. Cem. Concr. Res..

[28] Ye G., Van Breugel K., Fraaij A.L.A. (2001). Experimental study on ultrasonic pulse velocity evaluation of the microstructure of cementitious material at early age. Heron.

[29] Jalal M. (2012). Durability enhancement of concrete by incorporating titanium dioxide nanopowder into binder. J. Am. Sci..

[30] Jalal M., Fathi M., Farza M. (2013). Effects of fly ash and TiO_2_ nanoparticles on rheological, mechanical, microstructural and thermal properties of high strength self compacting concrete. Mech. Mater..

[31] Lawrence P., Cyr M., Ringot E. (2003). Mineral admixtures in mortars-Effect of inert materials on short-term hydration. Cem. Concr. Res..

[32] Jayapalan A., Lee B., Kurtis K., Bittnar Z., Bartos P.J.M., Nemecek J., Smilauer V., Zeman J. (2009). Effect of Nano-sized Titanium Dioxide on Early Age Hydration of Portland Cement. NICOM3.

[33] Folli A., Pade C., Hansen T.B., De Marco T., Macphee D.E. (2012). TiO_2_ photocatalysis in cementitious systems: Insights into self-cleaning and depollution chemistry. Cem. Concr. Res..

[34] Chen J., Kou S.-C., Poon C.-S. (2012). Hydration and properties of nano-TiO_2_ blended cement composites. Cem. Concr. Compos..

[35] Nazari A. (2011). The effects of curing medium on flexural strength and water permeability of concrete incorporating TiO2 nanoparticles. Mater. Struct..

[36] Noorvand H., Ali A.A.A., Demirboga R., Farzadnia N., Noorvand H. (2013). Incorporation of nano TiO_2_ in black rice husk ash mortars. Constr. Build. Mater..

[37] Li H., Zhang M.-H., Ou J.-P. (2006). Abrasion resistance of concrete containing nano-particles for pavement. Wear.

[38] Zhang M.-H., Li H. (2011). Pore structure and chloride permeability of concrete containing nano-particles for pavement. Constr. Build. Mater..

[39] Soleymani F. (2012). The filler effects TiO_2_ nanoparticles on increasing compressive strength of limestone aggregate-based concrete. J. Am. Sci..

[40] Rashad A.M. (2015). A Synopsis about the Effect of Nano-Titanium Dioxide on Some Properties of Cementitious Materials-a Short Guide for Civil Engineer. Rev. Adv. Mater. Sci..

[41] Meng T., Yu Y., Qian X., Zhan S., Qian K. (2012). Effect of nano-TiO_2_ on the mechanical properties of cement mortar. Constr. Build. Mater..

[42] Lee B.Y. (2012). Effect of Titanium Dioxide Nanoparticles on Early Age and Long Term Properties of Cementitious Materials. Ph.D. Thesis.

[43] Behfarnia K., Keivan A., Keivan A. (2013). The effects of TiO_2_ and ZnO nanoparticles on physical and mechanical properties of normal concrete. Asian J. Civ. Eng..

[44] Leslie J., Cheesman W. (1949). An ultrasonic method of studying deterioration and cracking in concrete structures. J. Am. Concr. Inst..

[45] Lee H.K., Lee K.M., Kim Y.H., Yim H., Bae D.B. (2004). Ultrasonic in-situ monitoring of setting process of high-performance concrete. Cem. Concr. Res..

[46] Robeyst N., Gruyaert E., Grosse C.U., De Belie N. (2008). Monitoring the setting of concrete containing blast-furnace slag by measuring the ultrasonic p-wave velocity. Cem. Concr. Res..

[47] Gallegos D.P., Munn K., Smith D.S., Stermer D.L. (1987). A NMR Technique for the Analysis of Pore Structure: Application to Materials with Well-Defined Pore Structure. J. Colloid Interface Sci..

[48] Schreiner L.J., MacTavish J.C., Miljković L., Pintar M., Blinc R., Lahajnar G., Lasic D., Reeves L.W. (1985). NMR Line Shape-Spin-Lattice Relaxation Correlation Study of Portland Cement Hydration. J. Am. Ceram. Soc..

[49] McDonald P.J., Korb J.P., Mitchell J., Monteilhet L. (2005). Surface relaxation and chemical exchange in hydrating cement pastes: A two-dimensional NMR relaxation study. Phys. Rev. E.

[50] Hürlimann M.D. (1998). Effective gradients in porous media due to susceptibility differences. J. Magn. Reson..

[51] Valckenborg R.M.E. (2001). NMR on Technological Porous Materials.

[52] Taylor H.F. (1997). Cement Chemistry.

[53] Allen A.J., Thomas J.J., Jennings H.M. (2007). Composition and density of nanoscale calcium-silicate-hydrate in cement. Nat. Mater..

[54] (2005). EUROPEAN STANDARD NORME196 Part 3, Methods of testing cement. Determination of Setting Times and Soundness.

[55] Karakosta E., Diamantopoulos G., Katsiotis M.S., Fardis M., Papavassiliou G., Pipilikaki P., Protopapas M., Panagiotaras D. (2010). In Situ Monitoring of Cement Gel Growth Dynamics. Use of a Miniaturized Permanent Halbach Magnet for Precise 1H NMR Studies. Ind. Eng. Chem. Res..

[56] Provencher S.W. (1982). A Constrained Regularization Method for Inverting Data Represented by Linear Algebraic or Integral Equations. Comput. Phys. Commun..

[57] Parrott L.J., Geiker M., Gutteridge W.A., Killoh D. (1990). Monitoring Portland cement hydration: Comparison of methods. Cem. Concr. Res..

[58] Tsivilis S., Kakali G., Chaniotakis E., Souvaridou A. (1998). A study on the hydration of Portland limestone cement by means of TG. J. Therm. Anal. Calorim..

[59] Cioffi R., Marroccoli M., Santoro L., Valenti G. (1992). DTA study of the hydration of systems of interest in the field of building materials manufacture. J. Therm. Anal. Calorim..

[60] Seki S., Kasahara K., Kuriyama T., Kawasumi M. Effects of Hydration of cement on compressive strength, modulus of elasticity and creep of concrete. Proceedings of the Fifth International Symposium on the Chemistry of Cement.

[61] Ye G., Lura P., van Breugel K., Fraaij A.L.A. (2004). Study on the development of the microstructure in cement-based materials by means of numerical simulation and ultrasonic pulse velocity measurement. Cem. Concr. Res..

[62] Keating J., Hannant D.J., Hibbert A.P. (1989). Correlation between cube strength, ultrasonic pulse velocity and volume change for oil well cement slurries. Cem. Concr. Res..

[63] Gaunaurd G., Überall H. (1981). Resonance theory of bubbly liquids. J. Acoust. Soc. Am..

[64] Feylessoufi A., Tenoudji F.C., Morin V., Richard P. (2001). Early ages shrinkage mechanisms of ultra-high-performance cement-based materials. Cem. Concr. Res..

[65] Bentz D.P., Coveney P.V., Garboczi E.J., Kleyn M.F., Stutzman P.E. (1994). Cellular automaton simulations of cement hydration and microstructure development. Model. Simul. Mater. Sci. Eng..

[66] Trtnik G., Turk G. (2013). Influence of superplasticizers on the evolution of ultrasonic P-wave velocity through cement pastes at early age. Cem. Concr. Res..

[67] Liu Z., Zhang Y., Jiang Q., Sun G., Zhang W. (2011). In situ continuously monitoring the early age microstructure evolution of cementitious materials using ultrasonic measurement. Constr. Build. Mater..

[68] Trtnik G., Valič M.I., Kavčič F., Turk G. (2009). Comparison between two ultrasonic methods in their ability to monitor the setting process of cement pastes. Cem. Concr. Res..

[69] Reinhardt H., Grosse C. (2004). Continuous monitoring of setting and hardening of mortar and concrete. Constr. Build. Mater..

[70] Buchwald A., Tatarin R., Stephan D. (2009). Reaction progress of alkaline-activated metakaolin-ground granulated blast furnace slag blends. J. Mater. Sci..

[71] Overloop K., Gerven L.V. (1992). NMR relaxation in adsorbed water. J. Magn. Reson..

[72] Blinc R., Lahajnar G., Zumer S., Pintar M.M. (1988). NMR Study of the Time Evolution of the Fractal Geometry of Cement Gels. Phys. Rev. B.

[73] Karakosta E., Lagkaditi L., ElHardalo S., Biotaki A., Kelessidis V.C., Fardis M., Papavassiliou G. (2015). Pore structure evolution and strength development of G-type elastic oil well cement. A combined 1 H NMR and ultrasonic study. Cem. Concr. Res..

[74] Lee B.Y., Kurtis K.E. (2010). Influence of TiO2 nanoparticles on early C3S hydration. J. Am. Ceram. Soc..

[75] Bentz D.P., Garboczi E.J. (1991). Percolation of phases in a three-dimensional cement paste microstructural model. Cem. Concr. Res..

[76] Scherer G.W., Zhang J., Quintanilla J.A., Torquato S. (2012). Hydration and percolation at the setting point. Cem. Concr. Res..

[77] Gussoni M., Greco F., Bonazzi F., Vezzoli A., Botta D., Dotelli G., Sora I.N., Pelosato R., Zetta L. (2004). 1H NMR spin-spin relaxation and imaging in porous systems: An application to the morphological study of white portland cement during hydration in the presence of organics. Magn. Reson. Imaging.

